# A 4K-Input High-Speed Winner-Take-All (WTA) Circuit with Single-Winner Selection for Change-Driven Vision Sensors

**DOI:** 10.3390/s19020437

**Published:** 2019-01-21

**Authors:** Fernando Pardo, Càndid Reig, José A. Boluda, Francisco Vegara

**Affiliations:** 1 Department of Computer Engineering, E.T.S.E., Universitat de València, Avd. de la Universidad, s/n, 46100 Burjassot, Spain; Jose.A.Boluda@uv.es (J.A.B.); Francisco.Vegara@uv.es (F.V.); 2 Department of Electronic Engineering, E.T.S.E., Universitat de València, Avd. de la Universidad, s/n, 46100 Burjassot, Spain; candid.reig@uv.es

**Keywords:** Winner-Take-All (WTA), Selective Change Driven Vision (SCD)

## Abstract

Winner-Take-All (WTA) circuits play an important role in applications where a single element must be selected according to its relevance. They have been successfully applied in neural networks and vision sensors. These applications usually require a large number of inputs for the WTA circuit, especially for vision applications where thousands to millions of pixels may compete to be selected. WTA circuits usually exhibit poor response-time scaling with the number of competitors, and most of the current WTA implementations are designed to work with less than 100 inputs. Another problem related to the large number of inputs is the difficulty to select just one winner, since many competitors may have differences below the WTA resolution. In this paper, a WTA circuit is presented that handles more than four thousand inputs, to our best knowledge the hitherto largest WTA, with response times below the microsecond, and with a guaranty of just a single winner selection. This performance is obtained by the combination of a standard analog WTA circuit and a fast digital single-winner selector with almost no size penalty. This WTA circuit has been successfully employed in the fabrication of a Selective Change-Driven Vision Sensor based on 180 nm CMOS technology. Both simulated and experimental results are presented in the paper, showing that a single pixel event can be selected in just 560 ns, and a multipixel pixel event can be processed in 100 μs. Similar results with a conventional approach would require a camera working at more than 1 Mfps for the single-pixel event detection, and 10 kfps for the whole multipixel event to be processed.

## 1. Introduction

The selection of the most relevant data is a key process in many computational strategies based on data-driven execution. A good selection strategy optimizes the use of computing resources. In many cases, it also facilitates their compression by eliminating redundant data. Many selection strategies can be found in different computational models involving competitive stages. One of the simplest and most extensively used is the so-called winner-take-all (WTA) [1], which is defined as a function ${\mathrm{WTA}}_{n}:{\mathbb{R}}^{n}\to {\left\{0,1\right\}}^{n}$ whose output $\langle b_{1},\;\ldots ,\;b_{n}\rangle ={\mathrm{WTA}}_{n}\left(x_{1},\ldots ,\;x_{n}\right)$ satisfies:

(1) $$b_{i}=\{\begin{array}{llll} 1, & \mathrm{if} & x_{i}>x_{j} & \mathrm{for}\;\mathrm{few}\;j\neq i\\ 0, & \mathrm{if} & x_{i}<x_{j} & \mathrm{for}\;\mathrm{the}\;\mathrm{most}\;j\neq i \end{array}$$

Such a definition is somehow ambiguous in most of the cases, so common variations of the WTA are often considered such as: single/hard WTA, where a single output bit *b_i_* has a value of 1, marking the position of the largest input *x_i_*; *k*-WTA, where the *i*th output *b_i_* has a value 1 if, and only if, *x_i_* is among the *k* largest inputs; or soft WTA, where the *i*th output is an analog variable *r_i_* whose value reflects the rank of *x_i_* among the input variables. It should be noted that Loser-Take-All (LTA) strategies can also be defined in a similar way. WTA based computational modules have long been implemented in, for example, artificial neural networks and active vision.

The simplicity and power of the WTA concept also allows the implementation of WTA-based circuits in high-performance analog electronic applications involving competing processes, such as bioinspired (neuromorphic) vision sensors [2,3]. In this new paradigm of vision sensors, images are not managed as frames, but as events, requiring specific electronic circuitry to process, in real time, this nonconventional representation of pixels. Within this framework, Selective Change-Driven (SCD) sensors have emerged as promising vision sensors taking advantage of the characteristics of WTA circuits within the Address Event Representation (AER) approach. SCD vision is a sensing strategy that aims at the prioritized selection of visual information attending to its relevance [4]. An SCD vision sensor delivers the pixel that has undergone the largest change in illumination since the last time that pixel was read out. It is possible to consider that the pixel showing the largest change is probably the best one to be processed at each time, since it probably has the largest contribution to the vision-processing task being performed. The (single/hard) WTA circuit included in these SCD sensors is key in order to select the best pixel to read at any instant.

## 2. State of the Art

WTA circuit performance is conditioned by three major parameters: resolution, time response, and scalability. These parameters are related to each other and have a close dependence on the number of inputs or competitors. Response time usually increases linearly with the number of competitors, since they usually share a single node for comparison purposes. Regarding WTA resolution (the capability to separate competitors with similar magnitude), a high number of inputs increases the probability of identifying more than one winner with differences below the resolution, which is a handicap in SCD sensors. As a consequence, there is room for improvement in the scalability of WTA circuits, still requiring research efforts.

Different efficient implementations of WTA in analog VLSI have been reported, starting with Reference [5]. This pioneer circuit provides computation for an approximate version of WTA with *n* inputs, with just 2*n* transistors and wires of length O(*n*). Since then, different approaches have been increasingly proposed to improve the aforementioned characteristics. Some selected representative contributions were collected in Table 1, along with their main figures.

In Reference [6], a first analysis on scalability was performed by reporting on the dependence of the response time against the number of cells in a WTA with a cascoded stage. Although simulations are carried out for a 1024 input circuit, experimental data are only provided for an arrangement of four chips with 50 inputs per chip. The gain (and then the resolution) was improved in Reference [7] by using a gain-boosted regulate-cascoding technique in a basic current-mode WTA circuit. With the aim of increasing speed and resolution, inhibitory and excitatory feedback stages, based on input currents’ average computation, were introduced in Reference [8]. In this case, 12 transistors are required per cell (two were required in the initial approach [5]). Only simulation results are reported for eight cell circuits. A slightly different approach was presented in Reference [9], where authors focused their efforts on the design of a programmable multiwinner k-WTA for low-voltage (*V_DD_* = 1 V) applications. Such a scheme requires the use of comparators and a negative-voltage generator, which increases the complexity of the circuit. Simulation results are obtained for an eight-cell WTA. A quantitative step regarding the number of managed inputs was introduced in Reference [10] by our own group, where a mixed analog/digital WTA circuit was designed and fabricated for processing the signals of a 1k pixel vision sensor. From a different point of view, maximum and minimum circuits (analog output) are described in Reference [11] based on WTA strategy. Simulation results for a two-cell case are reported. The improvement of the gain is revisited in Reference [12], where the use of an additional stage is introduced to the Lazzaro cell. The performance of the approach is experimentally demonstrated for a four-input circuit. On the other hand, in Reference [13], improvement on the dynamic range was approached by means of a modified Lazzaro’s cell. Nevertheless, the authors focused their study on three-cell analog applications. In Reference [14], voltage buffers were incorporated in the WTA circuit in order to reduce the bias voltage to 0.5 V.

From prior analysis, it can be concluded that major research efforts have been dedicated to enhance the gain of WTA circuits and their resolution by considering additional stages at the price of enlarging complexity and the required silicon area. On the other hand, the best dynamic responses have only been demonstrated for WTA circuits with few inputs. In this paper, an improved WTA circuit is presented. This novel approach consists of two blocks: (i) an analog WTA circuit, optimized for a large number of inputs, and (ii) a digital selector, resolving the single winner from the set of potential ones given by the analog WTA. Between both main blocks, a biasing/comparator interface is placed in order to optimize winner identification. The complete circuit was implemented in standard 180 nm CMOS technology within the design of an SCD sensor [15,16] with 64 × 64 pixels (resulting in 4096 inputs for the WTA circuit). Performance in terms of resolution, stability, time response, and scalability of WTA circuits is analyzed and discussed.

## 3. Winner-Take-All Scheme Description

### 3.1. Winner-Take-All Analog Circuit

**Figure 1:**
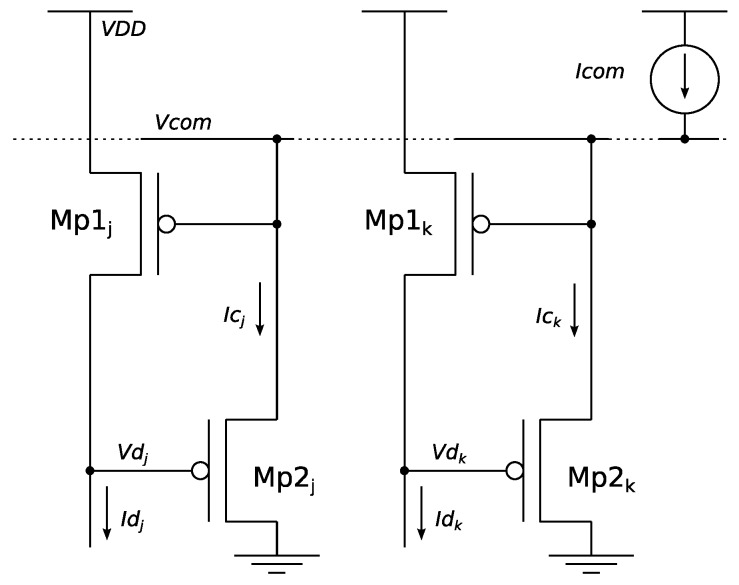
WTA circuit proposed by Lazzaro.

The basic (analog) WTA circuit, as firstly proposed by Lazzaro [5] is depicted in Figure 1 for a two-cell (*j* and *k*) pMOS (saturated) configuration. In a real scenario, *Id_j_* and *Id_k_* should be the input signals codifying the particular magnitude to be processed. A quiescent state in which *Id_j_* = *Id_k_* is initially assumed. Ideally being that Mp1*_j_* = Mp1*_k_* and Mp2*_j_* = Mp2*_k_*, *Ic_j_* = *Ic_k_* = *I*com/2 is induced. As a consequence, *Vd_j_* = *Vd_k_*. If, eventually, *Id_j_* current increases by a small amount of *δI*, Mp1*_j_* sinks this extra current by increasing its *V_DS_* voltage, and then it decreases drain voltage *Vd_j_* due to the Early effect. Subsequently, *V_GS_* in Mp2*_j_* increases, promoting the rise of *Ic_j_*. Because *I*com is necessarily equal to *Ic_j_* + *Ic_k_*, the increase of *Ic_j_* produces a reduction of *Ic_k_*, which forces a decrease of *Vd_k_*, again due to the Early effect, while keeping *Id_k_* constant. This process has a positive feedback, so that at the end *Ic_j_* is close to *I*com, *Id_k_* close to zero, *Vd_j_* the lowest allowed by the surrounding device, and *Vd_k_* close to *V_DD_*.

Such a basic configuration has well-known constraints related to resolution, speed, and scaling. Regarding resolution, when the change in *Id_j_* (resolution *δI*) is not large enough for shooting the winning process, it is possible to find a nonwinning cell with *Id_k_* still large enough to be incorrectly considered as a winner. This situation is particularly common in WTA circuits with many inputs, where a large set of cells may have very similar winning currents. By improving the resolution, the number of winners is reduced. The resolution of this WTA circuit can be improved if the excitatory and inhibitory input stages are considered as described in Reference [8]. An excitatory stage increases the input current of winner *Id_j_*, while an inhibitory stage decreases the input current of the losers. In this way, the resolution of the WTA is notably improved at the price of requiring a reset step after every competition process. For many input systems, significant delay is added, which can often be unacceptable.

Resolution of WTA analog circuits can also be improved by increasing the gain of the input stage by using, for example, cascode elements [7,17]. Such approaches provide better resolutions, but they cause circuit instability due to the capacitance of the *V*com node. Since this capacitance increases with the number of inputs, this approach is better for few-input scenarios or with moderate gains.

The capacitance of the *V*com node is also in the origin of the scaling limitations of WTA circuits. This capacitance grows linearly with the number of competing cells, introduces instability at the output of each cell (*V_d_*), and slows down the global WTA response. Nevertheless, because most of the main processes take place within a particular cell, speed is only barely conditioned by the number of competing cells.

**Figure 2:**
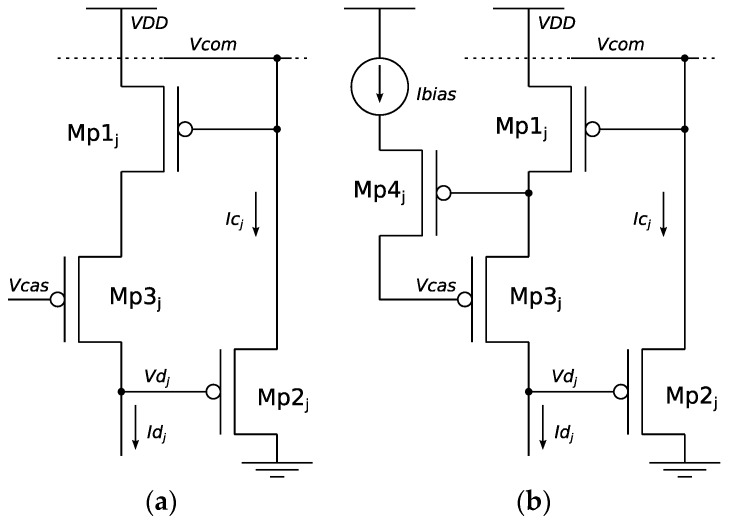
(**a**) Cascode WTA [5]. (**b**) Boosted-cascode WTA [7].

As a trade-off between resolution, stability, and speed, it has been considered an improved-gain input stage, as is depicted in Figure 2a. It is based on the addition of a cascoded transistor (Mp_3_) between Mp_1_ and Mp_2_, as proposed in Reference [17]. This way, output resolution is increased in about the order of the output impedance increase introduced by Mp_3_, which is about one order of magnitude. When considering higher gain stages, such as that proposed in Reference [7] and depicted in Figure 2b, unacceptable oscillations appear. As is demonstrated later, with the proposed circuit, the obtained resolution is appropriated for avoiding instabilities, but is not high enough for avoiding multiwinner situations. This multiwinner issue is solved in Section 3.3.

### 3.2. Winner Identification

Any winner *j* can be identified by the voltage at WTA node *Vd_j_*. Winner pixels have lower *Vd_j_* voltage than losers, which should have voltage near to *V_DD_*. The *Vd_j_* voltage of the winners depends on the number of potential winners (the fewer the winners, the lower the voltage) and the comparing current *Id_j_* (the higher the current, the lower the voltage). By introducing an adjustable threshold, it is possible to distinguish winners from losers. Moreover, by adjusting this threshold, it is possible to modify the number of total selected winners.

**Figure 3:**
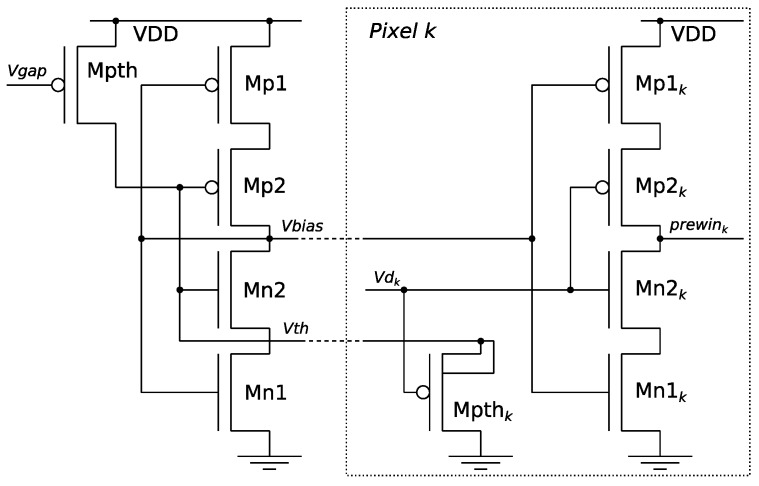
Adjustable comparator for winner identification.

Figure 3 shows the proposed adjustable-threshold circuit based on the voltage comparator described in Reference [7]. The inverter, formed by Mp1, Mp2, Mn2 and Mn1, generates the common *V*_bias_ to the comparators of all the pixels in the array. The figure also shows the comparator of one of the pixels (*k*); its output prewin*_k_* indicates whether it is a potential winner (prewin*_k_* = 1) or not. The comparator of each pixel (slave) is identical to the bias generator (master); thus, for the same *V*_bias_, they have the same comparing threshold *V*_th_. To ensure that at least one winner is selected, a value of *V*_th_ was chosen that is always above the minimum *V_dk_* in the array. This is done by means of transistors Mpth and Mpth*_k_* as follows: let us suppose pixel *k* is a clear winner with the lowest *Vd_k_*. In this case, any Mpth*_j_* (*j* = *k*) is cut off and the drain current of Mpth*_k_* is the same of Mpth; thus, the *V_gs_* of both transistors are the same. *V_gs_* of Mpth is adjustable value *V*_gap_, and *V_gs_* of Mpth*_k_* is *Vd_k_* – *V*_th_. Therefore, *V*_th_ = *V_dk_* + *V*_gap_, that is to say, comparing threshold voltage *V*_th_ is always above the voltage of winner *Vd_k_* by an adjustable amount of *V*gap. In the case of several winners, the current of Mpth is shared among the winners and *Vd_k_* < *V*_th_ < *Vd_k_* + *V*_gap_. This is a good behavior because it is better to approximate the comparing threshold to the voltage of the potential winners to reduce the number of selected winners when there are many. *V*_gap_ is an input of the sensor that is set externally and serves to control the number of potential winners.

### 3.3. Digital Single-Winner Selection

It has been shown in the previous section that the worst case in a WTA circuit occurs when there is a single winner and it loses because there is delay in selecting a new winner that directly depends on the number of competitors. It is then a good idea to always have a set of several winners from the analog WTA instead of just one winner. For a vision sensor, this is not a big problem because it almost makes no difference to select exactly the best pixel or just one of the best ones. This is the reason to adopt Lazzaro’s WTA with the moderate increase of resolution introduced by cascode configuration. This is also the reason why WTA circuits with input excitatory stages have been avoided, since each election implies slow global competition.

The digital single-winner stage selects one single winner from the set of potential winners calculated by the analog WTA circuit. It must be fast and scalable. A previous digital single-winner selector implemented in Reference [2] was based on a single-priority path: all pixels were connected in a pipeline forming a priority list. Each cell had an inhibition input and output, the inhibition output of one cell being connected to the inhibition input of the next. If the inhibition input of a cell is active, the cell activates the inhibition output to the next. If a cell detects that it is a potential winner and the inhibition input is not active, it signals itself as the only winner. Otherwise, it does not consider itself as a winner. In both cases, it activates the inhibition output. This simple mechanism ensures that there is only one winner among potential winners. The transmission of the inhibition signal is digital and very fast, but it has to traverse all the cells in the array and its delay is O(n). While this is fine for tens of pixels, it is not tolerable for thousands.

The proposed mechanism in this paper is different and its delay is $O\left(\sqrt{n}\right)$, because it uses a bidimensional priority mechanism that selects a single winner in two phases: the first selects a single column with winners, and the second, a single row with the winner of that column.

**Figure 4:**
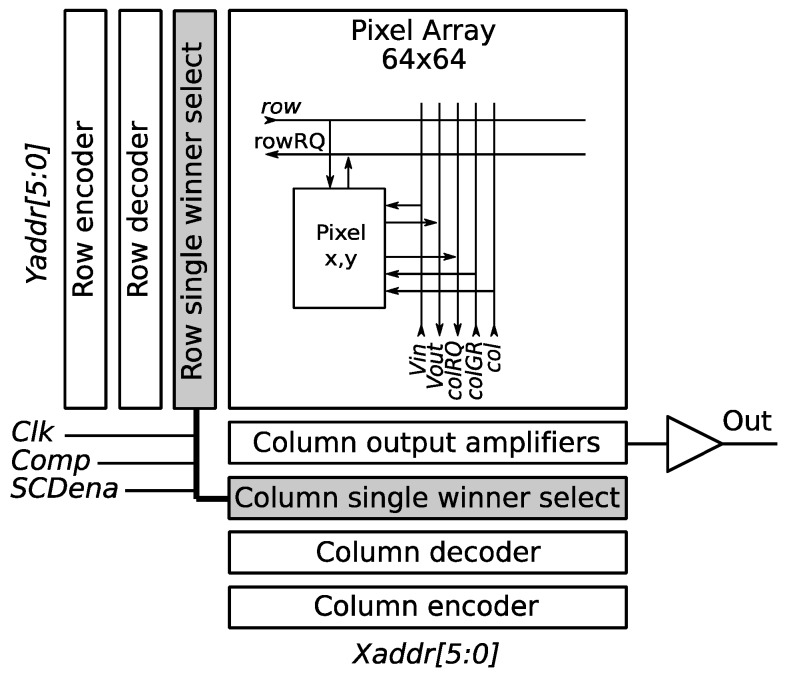
Sensor blocks with the digital single-winner selection circuits and signals.

Figure 4 shows the blocks of the SCD sensor. The blocks’ Column single-winner select and Row single-winner select form the digital single-winner selection circuit. Inside of the pixel array of this figure, a single pixel is represented with its column and row signals. Any potential winner of the array (there can be many as selected by the analog WTA) sets its Column Request (colRQ) signal. The 64 colRQ signals enter the Column single-winner select circuit, which is an inhibitory pipeline as stated before, with the difference that it only has 64 elements instead of all the pixels. This circuit selects just one column asserting the Column Granted (colGR) signal back to the pixel in the array. All the winner pixels in that column then assert the rowRQ signal to the Row single-winner select circuit that selects just one of them. At that moment, the decision for the winner pixel is taken, but it is not read out until it is externally indicated by the Clk signal. When the Clk is high, the row and column of the winner pixel are latched to the corresponding row and col signals. In this way, the pixel with row and col activated knows that it is the winner. Then, it opens its output buffer, resetting the internal capacitor that stores the last read-out value. Since the current and last illumination levels are now equal, its difference is zero, and this pixel immediately loses the competition, initiating new winner selection. The winner pixel remains selected by col and row until the Clk signal is again set, and a new pixel is selected. The 64-bit row and col signals are encoded to generate the 6-bit *Y* and *X* addresses, respectively, of the winner pixel. An external processor can take the address and the illumination level of the winner pixel to process them. This is a normal SCD operation, where pixels are read out ordered by their illumination-level change. However, this sensor is also prepared to work as a frame-based standard CMOS vision sensor. In that case, the address is provided by an external processor to the sensor in order to select the desired pixel. The internal Row and Column decoders translate the address to a specific row and column that select the pixel to read out. The operation mode is selected with the SCDena signal (SCD enable).

The analog WTA competition for the next pixel takes place while the current pixel is being read out. During the digital selection, the rowRQ, colRQ, and colGR may change many times, generating noise in the analog signals. To avoid this, digital selection is only enabled at the capture time of the winner pixel using the Competition (Comp) signal; thus, Comp and Clk have a similar waveform, and the only difference is that the Comp high pulse is 20 ns wider.

## 4. Simulation Results

The SCD sensor was not designed to access the internal nodes of each WTA cell, but it is still possible to perform global physical experiments, as it is shown in next section, that hold some of the simulated results presented in this section. It was discussed in the previous sections that the promotion of a pixel to become a winner is a local process, as well as the degradation from winner to loser when there is more than one winner selected; thus, these processes are almost independent of cell number and they scale well with the WTA inputs. Fortunately, this is the normal situation in an SCD vision sensor with thousands of pixels. The following simulated experiment shows this behavior.

**Figure 5:**
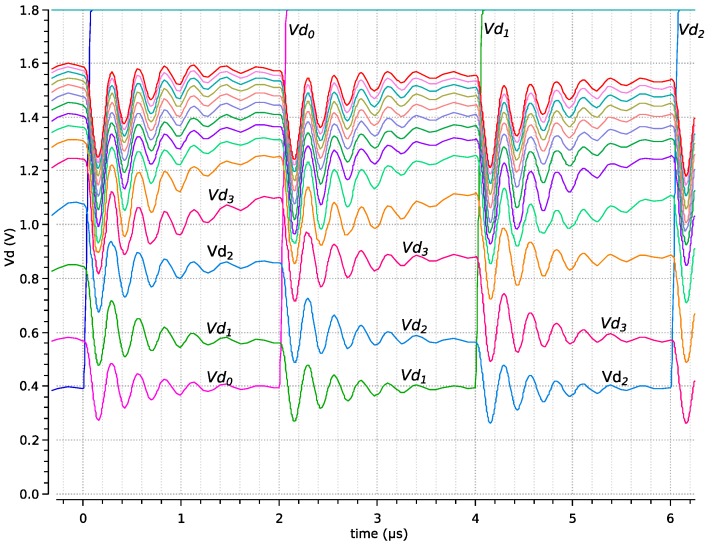
Normal WTA operation of successive losing pixels.

A simulation was performed supposing a camera moving in an artificially illuminated environment. In this situation, many pixels change at the same time. The pixels that have changed the most have a similar change, although slightly different. Figure 5 shows a simulation with the 4K (64 × 64) pixel array of the implemented SCD sensor. The *Vd_i_* voltage of the 14 pixels with the largest change is shown in the figure. Every 2 µs, a pixel is readout, and its *Id* current, which is proportional to the illumination change, becomes zero. The *Vd* of the selected pixel almost instantly rises to *V_DD_* (+1.8 V), and a new pixel can be selected to be the next winner. This process is shown in the figure: at the beginning (time = 0) Pixel 0 is the pixel with the lowest *Vd*; thus, at time = 2 µs, it is selected as the winner, its *Id*_0_ is reset, and *Vd*_0_ rises to *V_DD_* in about 70 ns. At this time, all other *Vd_i_* readjust themselves, and Pixel 2 becomes the pixel with the lowest *Vd*. This process is continuously repeated at the rate fixed by external clock Clk. There is a small attenuated oscillation of the *Vd* signal that depends on transistor dimensions and the WTA number of inputs (the largest, the worst). The winner-comparison threshold is chosen to be above the amplitude of the oscillation to avoid the lack of winner selection.

**Figure 6:**
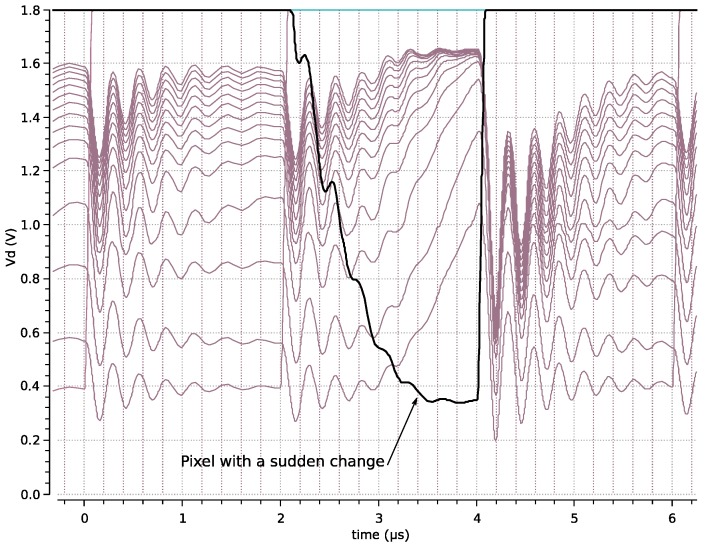
WTA delay when there is a large change in the illumination of one pixel and when it is promoted to be a winner.

Let us now suppose that one pixel suddenly registers a large change in illumination, for example, because of a bright spot has moved to that pixel. Figure 6 shows the simulation of such an event occurring at time = 2.1 µs. It is possible to see that it takes around 1 µs to become the winner. This time depends on many factors, including the number of inputs, but even with a large number like 4K inputs, this time is already below the period of the pixel rate. In fact, the response time of the photodiode employed in the pixel only reaches the microsecond delay in highly illuminated environments (mid-day outdoor sunny scenes and above) [3]. In normal illumination conditions, the WTA reacts faster than the photodiode itself.

**Figure 7:**
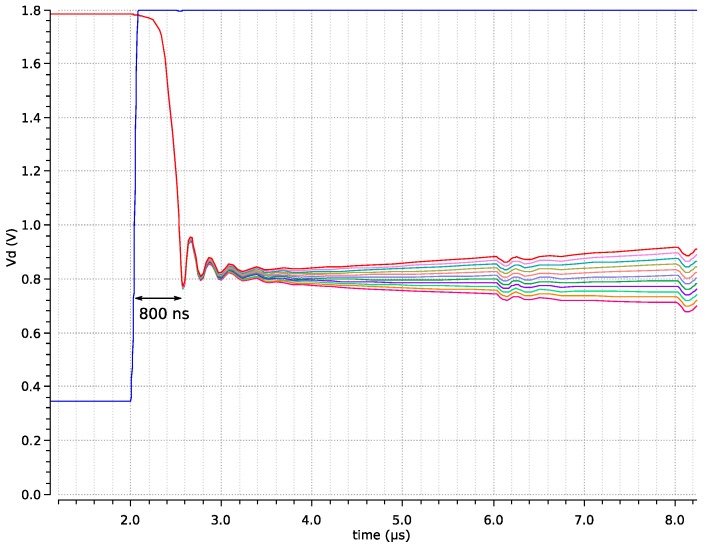
Worst case when there is a clear single winner, it is selected, and all other pixels fight to become the winner.

It has been shown that, in standard working conditions, becoming a winner is a very fast process, even with a large number of inputs, because there are always several potential winners from which to choose. One of the worst cases is the one already commented, of one or few pixels with a high sudden change, as can be seen in Figure 6. The worst case, however, probably happens when there is only one winner that is very different from the others, which immediately loses after it is selected. In this case, the *V*com node must adapt itself to the currents of new potential winners, and this takes time because this is a common node, of which the capacitance directly depends on the number of pixels. Figure 7 shows what happens when there is a clear winner that loses after being selected. The delay time before having new winners is in the order of one microsecond. This is roughly the same delay found in the case of a pixel being promoted to a winner when there already are some winners. The difference is that, during this delay, there are no winners from which to select. Taking into account that the normal pixel period of this sensor is 2 µs, this worst case has no impact in the output, though it could become noticeable at higher pixel rates.

## 5. Experimental Results

Some experiments have been carried out to demonstrate the detailed characteristics obtained in simulations, especially regarding fast reactions to events. Moreover, there are some characteristics, like the persistence of events over time, that cannot be reliably simulated but can be measured with the real sensor. In this way, the elapsed time between any event and its signaling in real conditions have been studied. Moreover, an experiment was also prepared to show for how long an event can be stored in an SCD sensor.

### 5.1. Event Reaction Time

Event reaction time is the time between the origin of an event (say, the fast switching of an LED) and the moment this event is noticed by the sensor. In the case of the SCD sensor, this time depends on three factors:

1. Time between pixel reads (pixel rate). This time fixes the minimum time resolution of the system and, therefore, of the signaling of any event. If illumination output is needed, then at least 1 µs between pixels is necessary; the sensor may work at lower times if no illumination level is required.
2. WTA delay. This is the time discussed in the WTA simulation section that is required by the WTA to provide a single winner. It has also been seen in the simulations that it is below 1 µs, even in the worst-case scenario.
3. Light-transduction delay. This is the time required by the photodiode and amplification circuits to convert illumination to an electrical measurable magnitude. The cell employed in this sensor continuously translates light into voltage, and its delay depends on the illumination intensity. The use of light integration-based cells, which is a common strategy for frame-based cameras, has been discarded for its large delay, which is not good for event-based sensors. The delay of continuously reading based sensors ranges from microseconds to milliseconds, depending on light intensity. Delays in the order of microseconds can be obtained with light intensities in the order of direct sunlight, or direct light coming from an LED located a few centimeters from the sensor.

The aim of these experiments was to measure the WTA delay. In order to do this, it is necessary to reduce to a minimum the time between pixel reads and the photodiode response time. The minimum pixel period for this sensor is 2 µs for reliable illumination output. This time can be further reduced with the cost of illumination-level degradation. Current sensor driving hardware also imposes a constraint of 1.12 µs on pixel read time if an illumination level is required, and 560 ns if only the pixel address has to be provided. These times are in the order of WTA-simulated delays; therefore, the experiments give an estimation of WTA behavior.

The experiment setup consisted of a highly luminescent LED located 6 cm in front of the SCD camera. The idea was to switch the LED on and observe the impact of this sudden event on the output over time. Since the SCD sensor reacts to differences in illumination from the last time the cell was read out, it is necessary to previously read all the cells in the sensor; this sets the cell internal-memory capacitor to the background value, so any change in the scene fires the WTA. This operation is performed just by reading a single image using the frame mode of the SCD sensor. Immediately after this image is taken, 4000 pixels are read in SCD mode at a constant pixel rate with a period of 1.12 µs. The microcontroller that drives the SCD camera was programmed to switch the LED on after Pixel 500 of this 4000-pixel sequence.

**Figure 8:**
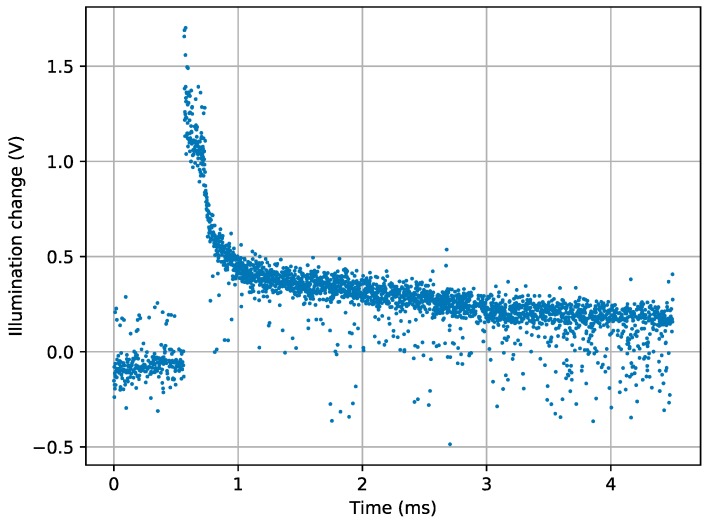
Illumination differences of 4000 pixels read in a period of 1.12 µs. The event (LED switching on) is introduced after Pixel 500 (0.560 ms).

Figure 8 shows the differences in illumination levels obtained for the 4000 pixels read in SCD mode when the LED is switched on after Pixel 500. Since the read out takes place with a period of 1.12 µs, which is the minimum allowed by the hardware, the event takes place at 0.560 ms. It is possible to see in the figure that the differences are roughly zero at the beginning and then they suddenly rise to a maximum that decreases faster at the beginning and slower after few pixels. This is exactly the behavior expected from an SCD sensor that first provides the pixels that have undergone the largest change, ordering the subsequent read-outs accordingly. There is a visible, though small, spread of the results mainly due to the mismatch of the WTA circuit, but it is not large and the figure shows a clear tendency to first read out the pixels with the largest change. Output spread is larger when there are many pixels with similar differences, and this happens when differences are smaller.

**Figure 9:**
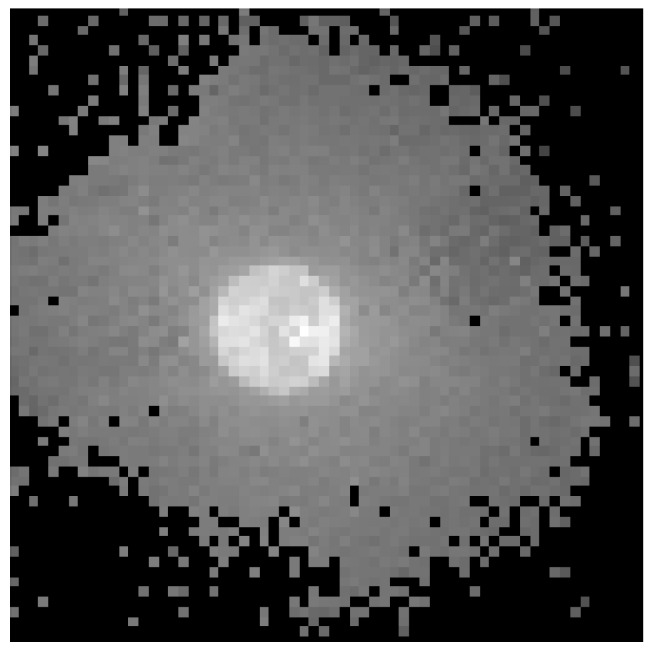
Reconstructed image showing the illumination level obtained with the fast LED switching experiment in Selective Change Driven (SCD) mode.

Figure 9 shows the “image” obtained from the pixels read in SCD mode. It is possible to see that few pixels correspond to the LED, while most of them correspond to the periphery. Comparing this image with Figure 8, it is easy to map the few pixels of the LED with the first pixels read after the event (from 0.560 to 0.750 ms in the figure). The rest of the pixels, meanwhile, belong to the LED “corona”, or periphery, formed by light dispersion around the LED. These periphery pixels are far more numerous, and they present similar illumination differences, though they can still be separated by the WTA circuit, as shown in Figure 8 from 1 ms to the end. There is a clear decreasing tendency in the curve despite the small illumination differences. These smaller illumination differences also produce higher dispersion and noise.

**Figure 10:**
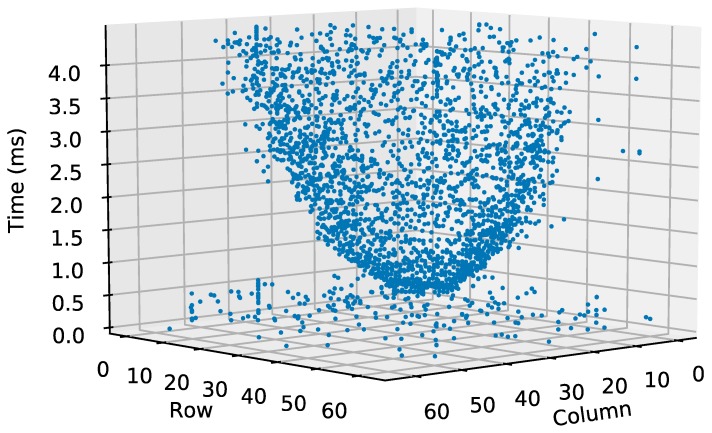
Three-dimensional representation of pixel co-ordinates (row and column) over time. Event occurs at 0.560 ms.

Figure 10 represents the pixel co-ordinates (row and column floor axes) over time (vertical axis). The figure shows an inverted cone, with its vertex at the precise moment the LED switches on. The cone vertex is wide because it includes most of the LED pixels that are quickly read out after the event happens. The rest of the cone corresponds to the LED corona pixels. This cone is formed because LED corona illumination slowly decreases from the LED center to the periphery. The WTA has enough resolution to still form a cone despite the small change in illumination in the corona.

**Figure 11:**
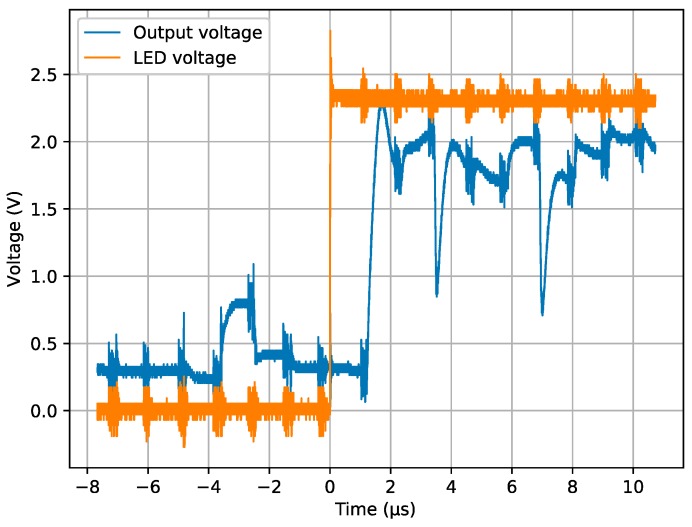
Output and LED voltages measured with a digital 1 GHz oscilloscope. Pixel-reading period is 1.12 µs.

Figure 8 and Figure 10 show good behavior of the WTA circuit in terms of resolution, but they provide little information about the delay between the event and the change at the output. Figure 11 shows the voltage applied to the LED and the sensor output, measured with a 1 GHz digital oscilloscope. Both signals show burst noise at regular intervals of 1.12 µs due to the sensor clocking for a pixel read out. The LED is immediately switched on after a pixel read on time t = 0 ms. It is possible to see that the very first read out, after the event has taken place, already corresponds to a highly illuminated pixel from the LED. The WTA was able to choose one of the pixels with the largest change in less than the smallest reading period that provides illumination output, which is 1.12 µs.

**Figure 12:**
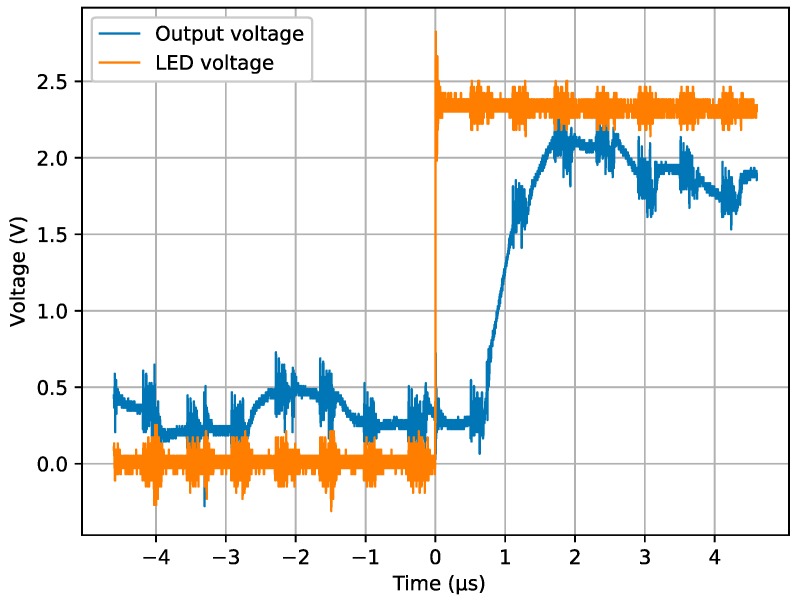
Output and LED voltages measured with a digital 1 GHz oscilloscope. Pixel-reading period was 560 ns.

The pixel-reading period can be reduced to 560 ns but, in this case, digitized illumination information is not available. The same experiment was repeated at this new pixel rate. Since illumination information is not available, it is not possible to plot illumination differences over time, but it is feasible to capture the analog output to see the time delay between event and output change. Figure 12 shows the output and LED voltages measured with the oscilloscope with a pixel interval of just 560 ns. In this case, like in the previous one, the next pixel read after the event is a highly illuminated pixel from the LED. The difference is that, as the interval between pixels is half of the previous case, it is possible to see how the analog output corresponding to the event is still changing. The reason is the delay in the photodiode circuit that inversely depends on the current created by LED intensity. This LED intensity, even at this short distance, is not enough to generate a sufficient current in the photodiode to produce a full change in less than 1 µs; this is the reason why it is possible to see this output still changing after the event. LED switching could also play a role in this delay but attending, to LED characteristics, full illumination should be accomplished in just tens of nanoseconds. In any case, WTA performance overcomes other hardware limitations of this SCD sensor, such as minimum pixel interval, photodiode speed, and real-scene events.

**Figure 13:**
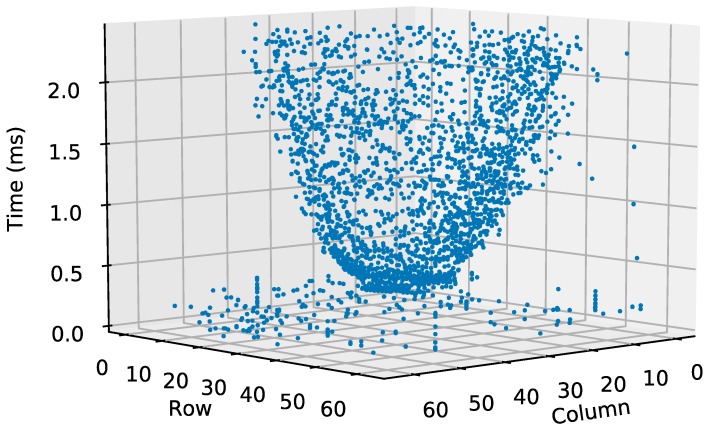
Three-dimensional representation of pixel co-ordinates (row and column) over time. Pixel-reading period was 560 ns. Event occurred after Pixel 500 (0.280 ms).

Although there is no illumination information, it is still possible to plot the co-ordinates of each pixel over time. Figure 13 shows the pixel co-ordinates as they are read out with a time interval of 560 ns. The result is very similar to that shown in Figure 10.

It is important to note that these experiments were performed by only using what is called “ON events”, that it is to say, events where illumination suddenly increases. These same experiments could also be carried out using OFF events (for example, by switching off the LED); the problem in this case is that the photodiode would take longer to register the change since it depends on illumination level. Delay OFF events, even in well-illuminated conditions, may have a delay of one or two orders of magnitude above the delay of ON events. This delay is far above the WTA performance already measured with ON events.

### 5.2. Event Retention Time

SCD sensors are based on the principle that pixels are ordered by the change that each pixel has undergone the last time they were read out. This principle does not impose restrictions on how far in time any event took place. The idea is that any event should eventually be processed, even if they have been delayed for long, due to the existence, for example, of many other events with higher priority. This behavior is one of the most important differences with respect to other event-based sensors, where events must immediately be processed or they are lost. SCD sensors use a capacitor in each cell to store the last read-out value and compare it with the current illumination level. This capacitor and the involved currents are very small, and they are difficult to simulate for obtaining a reliable result. Moreover, many factors can modify the stored value in the capacitor, such as noise, switching, and signal feedthrough. For most applications, just a few milliseconds or one second at most of retention should be enough, and it seems interesting to test for how long processing an event could be delayed in this SCD sensor.

**Figure 14:**
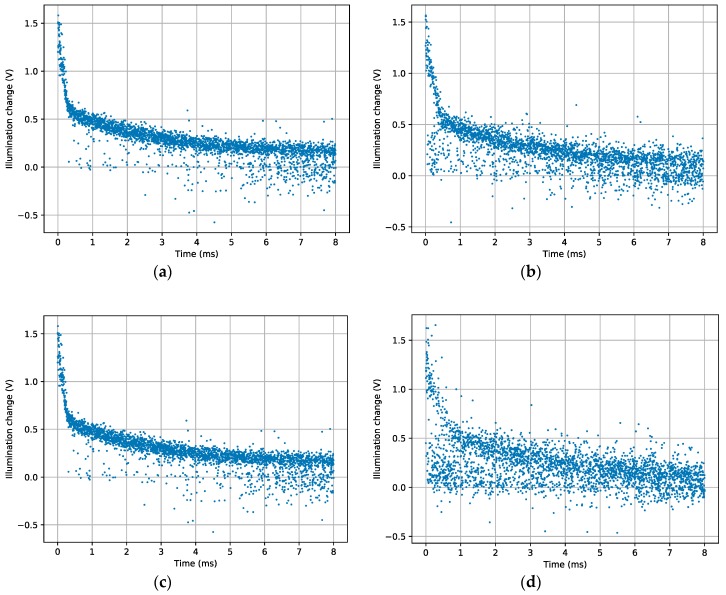
Illumination differences read with delays of (**a**) 0 s, (**b**) 10 s, (**c**) 20 s, and (**d**) 40 s after the event.

In order to test the capacity of this sensor to delay event processing, all pixels in frame mode were read to set all capacitors with the scene-background level. After that, the LED was switched on and the sensor was not accessed during different test times, from no delay to 40 seconds delay. Figure 14 shows the result of these experiments for delays of 0, 10, 20, and 40 s. In the case of 0 s delay (top-left figure), there is, in fact, a real delay of 200 ms; nonetheless, the obtained result is very similar to the already shown in Figure 8, which had no delay. The figure corresponding to a delay of 10 s after the event (top-right) shows slight output degradation, though most pixels still hold their difference. After 20 s (bottom-left), there were still many pixels that performed well, while after 40 s (bottom-right) most of the pixels were signaled in the wrong place. It is possible to conclude that the sensor performs really well at retaining the last read-out value, since pixels are usually accessed in much less than a second, even in the worst-case scenario.

## 6. Conclusions

A 4K input WTA circuit was designed and fabricated as part of an SCD sensor. The simulations and experiments performed with the WTA circuit showed good resolution for choosing a winner, and a delay of less than 1 µs, which is beyond the actual capacity of the SCD sensor. With this work, it was proven that it is possible to successfully use many input WTA circuits to prioritize the order in which pixels are read out from a sensor without losing performance. Events can be signaled and measured immediately after the next pixel read. In the experiments, it was shown that an event can be detected in just 560 ns, and a complete scene change, like the complete illumination of a LED, can be processed in the required time to read all the pixels of the LED (around 100 µs in this case). Compared to frame-based cameras, this time resolution could only be achieved with cameras working at more than 1 Mfps for first-event detection, or 10 kfps if it is necessary to wait for the whole LED to be processed. The processing hardware required by the SCD sensor was just a microcontroller working at 120 MHz, whereas the hardware required by a frame-based camera, working at already much lower speeds, such as 1000 fps, would be very complex.

## Figures and Tables

**Table 1:** Summary of the main performance parameters of previously published Winner-Take-All (WTA) circuits.

CMOS Technology	Number of Inputs	Number of Transistors	Time Delay	Resolution	Reference
2 μm (MOSIS)	170	2*n*	>100 μs	2%	[5]
2 μm (MOSIS)	200	10*n*	~300 ns	~50 mV	[6]
0.8 μm (AMS)	10	3*n*	n.p.	~1 nA	[7]
0.35 μm (TSMC)	8	12*n*	~15 ns	~2 nA	[8]
0.25 μm	8	7*n*	~50 μs	~5 mV	[9]
0.35 μm (AMS)	1024	4*n*	~2 μs	~10 nA	[10]
0.25 μm (TSMC)	2	3*n* + 4	n.p.	n.p.	[11]
0.5 μm (ON SEMI)	4	2*n* + 2	~1 μs	n.p.	[12]
0.13 μm	3	3*n* + 1	~50 ns	n.p.	[13]
0.18 μm	2	3*n* + 3	>1 μs	n.p.	[14]
0.18 μm (AMS)	4096	3*n* + 1	<1 μs	n.p.	This work.

## Abbreviations

The following abbreviations are used in this manuscript:

AER	Address Event Representation
CMOS	Complementary Metal Oxide Semiconductor
fps	Frames per second
kfps	Kiloframes per second
LED	Light-Emitting Diode
n.p.	Not provided
SCD	Selective Change-Driven
VLSI	Very Large Scale of Integration
WTA	Winner-Take-All
